# Coronavirus Disease 2019 (COVID-19)

**DOI:** 10.3390/biology11081250

**Published:** 2022-08-22

**Authors:** Mohamad Goldust

**Affiliations:** Department of Dermatology, University Medical Center Mainz, 55131 Mainz, Germany; mgoldust@uni-mainz.de

The coronavirus disease 2019 (COVID-19) pandemic has affected almost all aspects of daily life. The economic and social disruption has been devastating. However, it has also opened many doors to the new development of novel technologies, such as telemedicine. In this context, the editors of the journal *Biology* decided at the very beginning of this worldwide dilemma to create a Special Issue, entitled “Coronavirus Disease 2019 (COVID-19).” This Special Issue encompasses thirty-one articles covering various aspects of the current pandemic [1,2,3,4,5,6,7,8,9,10,11,12,13,14,15,16,17,18,19,20,21,22,23,24,25,26,27,28,29,30,31]. With contributions from esteemed scientists from across the globe, this Special Issue sheds light on various aspects of the pandemic, including, but not limited to, epidemiology, pathophysiology, risk factors, and possible treatment options. In summary, the key published articles in this Special Issue addressed the following topics. Firstly, the virus and its complex pathophysiology were evaluated in some of the published articles [3,4,5,8,22,29]. In addition, the importance of face masks and related subjects were highlighted in other studies [6,7]. Klugar and colleagues asserted the importance of vaccines and different types of vaccines in combatting the pandemic [10]. Rodriguez-Cerdeira et al. [17] and Conforti and his colleagues [18] demonstrated the cutaneous manifestations of SARS-CoV-2 and their major role in determining a better diagnosis of the disease. Finally, the management of COVID-19 was also highlighted in some of other published articles [1,13,19,28,30].

Special thanks goes to the strong international team of editors, including Prof. Robert A. Schwartz from the USA, Prof. Dedee F. Murrell from Australia, and Prof. Torello Lotti from Italy, for their immeasurable dedication and support.

We hope for universal cooperation in the use of appropriate vaccines and efforts to identify new and effective medications, so that the world might be free of SARS-CoV-2 in the near future and normal life might be resumed.
